# Annexin A1 protein regulates the expression of PMVEC cytoskeletal proteins in CBDL rat serum-induced pulmonary microvascular remodeling

**DOI:** 10.1186/1479-5876-11-98

**Published:** 2013-04-15

**Authors:** Bin Yi, Jing Zeng, Guansong Wang, Guisheng Qian, Kaizhi Lu

**Affiliations:** 1 Department of Anesthesia, Southwest Hospital, The Third Military Medical University, Chongqing 400038, China; 2 Institute of respiratory Disease, Xinqiao Hospital, The Third Military Medical University, Chongqing 400037, China

**Keywords:** Hepatopulmonary syndrome, Pulmonary microvascular endothelial cells, ANX A1

## Abstract

**Background:**

Hepatopulmonary syndrome (HPS) is characterized by advanced liver disease, hypoxemia and intrapulmonary vascular dilatation (IPVD). The pathogenesis of HPS is not completely understood. Recent findings have established the role of proliferation and phenotype differentiation of pulmonary microvascular endothelial cells (PMVECs) in IPVD of HPS; the change in cytoskeletal proteins and their molecular mechanism play an essential role in the proliferation, phenotype modulation and differentiation of PMVECs. However, little is known about the relevance of cytoskeletal protein expression and its molecular mechanism in IPVD of HPS. In addition, ANX A1 protein has been identified as a key regulator in some important signaling pathways, which influences cytoskeletal remodeling in many diseases, such as lung cancer, liver cancer, etc.

**Methods:**

PMVECs were cultured from the normal rats and then divided into three groups(Ad-ANXA1-transfected group, a non-transfected group, and an adenovirus empty vector group) and incubated by nomal rat serum or hepatopulmonary syndrome rat serum respectively. mRNA level was evaluated by real time reverse transcription polymerase chain reaction, and protein expression was detected by western blot. Cell proliferation was determined by the MTT and thymidine incorporation assay.

**Results:**

In this study, we found that the serum from a common bile duct ligation(CBDL) Rat model decreased the expression levels of the ANX A1 mRNA and protein by at least two-fold in human PMVECs. We also found the expression of cytoskeletal proteins (Destrin, a1-actin, and a1-tubulin) in PMVECs significantly increased. After stimulating ANX A1 over-expression in PMVECs by adenovirus-mediated ANX A1 (Ad-ANXA1) transfection, we found the expression levels of cytoskeletal proteins were significantly suppressed in PMVECs at all time points. Additionally, we report here that serum from a CBDL Rat model increases the proliferation of PMVECs by nearly two-fold and that over-expression of Ad-ANXA1 significantly inhibits HPS-rat-serum-induced PMVEC proliferation (*p* <0.05). These findings suggest that the ANX A1 down-regulation of PMVEC proliferation in the presence of HPS-rat-serum may be the major cause of aberrant dysregulation of cytoskeletal proteins (Destrin, a1-actin, and a1-tubulin) and may, therefore, play a fundamental role in the proliferation and phenotype differentiation of PMVECs in the PVD of HPS.

**Conclusion:**

Finally, the fact that transfection with Ad-ANXA1 results in inhibition of the aberrant dysregulation of cytoskeletal proteins and proliferation of PMVECs suggests a potential therapeutic effect on PVD of HPS.

## Introduction

Hepatopulmonary syndrome (HPS) is classically defined by a widened alveolar-arterial gradient at room temperature (>15 mmHg or >20 mmHg in patients >64 years of age), with or without hypoxemia resulting from intrapulmonary vasodilatation (IPVD) in the presence of hepatic dysfunction or portal hypertension [1]. HPS occurs in at least 15% of patients with end-stage liver disease but is thought to be widely underdiagnosed. Mortality within a year of diagnosis is markedly increased in HPS patients with cirrhotic compared to patients without cirrhotic. [2,3]. There is currently no effective medical treatment for HPS. Liver transplantation (LT) is considered the only dependable mode of therapy for HPS [4,5]. The main reason for the absence of effective therapeutic measures is that the pathophysiological mechanisms associated with HPS have not been identified.

Although not yet completely clear, the pathogenesis of HPS is believed to involve a number of factors. In particular, IPVD occurs in many cirrhotic patients and is believed to contribute to pulmonary microvascular remodeling and arterial hypoxemia [5,6]. Thus, IPVD and blunted hypoxic vasoconstriction were central to ventilation-perfusion mismatching, leading to arterial hypoxemia [7-9]. Currently, the hypotheses for explaining the pathogenesis of IPVD remain speculative, despite the various experimental studies that have been conducted. Emphasis has been given to the roles that many cytokines may play in pulmonary microcirculation and in the genesis of IPVD [10]. Following liver damage, endothelin (ET-1) produced in the liver migrates into pulmonary circulation and appears to interact preferentially with the ETB receptor, thereby promoting pulmonary vasodilatation [11]. Analysis of a model for liver cirrhosis and hyperdynamic circulation following common bile duct ligation(CBDL) revealed greater hepatic production and plasma circulation of ET-1, which is associated with the development of IPVD [12]. In cases of liver cirrhosis, the levels of tumor necrosis factor-alpha (TNF-α) also increase, which contributes to the accumulation of macrophages in the lumen of the pulmonary vessels. In turn, these macrophages stimulate another NO-producing enzyme (induced nitric oxide synthase, iNOS), thereby triggering IPVD [13]. These experimental findings regarding the roles of ET-1, TNF-α and other cytokines in the development of IPVD in pulmonary microvasculature may contribute to understanding the physiopathology of HPS in humans. However, treatment options aimed directly at cytokines, such as inhaled NG-nitro-L-arginine methyl ester (L-NAME), intravenous methylene blue, somatostatin, almitrine, indomethacin, norfloxacin, etc., have been tested in HPS cases, but currently no treatment offers satisfactory results for HPS reversion [13-17]. Further studies have been demanded and might offer a new approach to the therapeutic treatment of HPS.

Our previous data demonstrated that the proliferation of PMVECs plays an important role in the development of IPVD [18,19]. However, many signaling pathways, such as PI3K-Akt, MLCK, TGF-β/Smads, and PKC/ERK, are involved in the regulation of the differentiation or proliferation of PMVECs. These signaling pathways interact with each other, and blocking one pathway may not be sufficient to reduce the incidence and development of IPVD. The cytoskeleton is essential not only for the maintenance of cell shape and motility but also for the integration of cell signals that initiate and propagate alterations in these cellular properties. Because cytoskeletal proteins are very important in the proliferation and differentiation of PMVECs, we hypothesize that regulating the expression of cytoskeletal proteins may be a key upstream target and provide a new approach to HPS prevention and treatment. However, many uncertainties exist in this research area, including the relationship between cytoskeletal proteins and the proliferation or differentiation of PMVECs in HPS and the molecular mechanisms.

Annexin A1 (ANX A1) is a member of the calcium and membrane-binding family and plays an essential role in tumorigenesis and apoptosis. In recent years, there have been accumulating data indicating a novel role for ANX A1 in cell proliferation and migration. However, the role of ANX A1 in CBDL-rat-serum-induced PMVEC differentiation and proliferation has not been previously described.

A well-established experimental model for hepatopulmonary syndrome is biliary cirrhosis and portal hypertension in rats induced by CBDL [8] and is the only recognized model for the study of HPS [8].

The purpose of this work was to examine the role of cytoskeletal proteins in PMVEC responses to serum from the CBDL rat model and specifically to investigate the role of ANX A1 in regulating the expression of cytoskeletal proteins and in the proliferation and differentiation of PMVECs under CBDL-rat-serum conditions. Our data showed that the expression of cytoskeletal proteins and cell proliferation increased significantly following stimulation with CBDL rat serum. In addition, over-expression of Ad-ANXA1 provided potential mechanisms of protection.

## Materials and methods

### Reagents

The reagents used in the study included rabbit polyclonal anti-rat ANXA1 antibody (NeoMarkers,USA), rabbit polyclonal anti- rat a1-actin antibody (NeoMarkers,USA), rabbit polyclonal anti- rat a1-tubulin antibody (NeoMarkers,USA), rabbit polyclonal anti-rat destrin antibody (NeoMarkers,USA), Ad-ANXA1 (provided as a generous gift from the Institute of Respiratory Diseases, Xinqiao Hospital, Third Military Medical University), and immunohistochemistry kits (Boster, China).

### CBDL rat model, cell culture, grouping of PMVECs and Ad-ANXA1 transfection

All procedures performed on the rats were conducted according to the guidelines from the National Institutes of Health. The study protocol was approved by the committee on Animal Research of Southwest Hospital. Sprague Dawley rats (180–220 g body wt) were used in this study. The CBDL rat model has been built successfully in our initial study. Details of the surgery and postsurgical care were similar to the previous studies [8,20]. At PaO_2_ < 85 mm Hg, the blood serum of rats with P(A-a)O_2_ > 18 mm Hg and pulmonary vasodilatation (PVD) found on pathological sections was used for this study. Serum was separated from CBDL blood samples (centrifugation at 2000 × g/min in a Gyria for 10 min at 4°C) within 20 min of collection, was carefully handled to avoid hemolysis, and was stored at −80°C.

PMVECs were isolated from the rat lung as previously described [21]. Briefly, 2–5 g of rat peripheral tissue was isolated from a normal SD rat. Lung tissue was rinsed in 10 mM phosphate-buffered saline (PBS, pH 7.4), finely minced into small pieces and digested in 0.3% type II collagenase (Sigma Chemical Co., Oakville, Ontario) for 45 min at 37°C with occasional agitation. The digested suspension was filtered through 100 μm mesh, centrifuged at 500 × g and washed twice in PBS. The cell pellet was resuspended in binding buffer (2 μm Na-citrated, 1.2 μM NaH2PO4H2O, 5.6 μM Na2HPO4, 138.6 μM NaCl, and 1% bovine serum albumin) and incubated for 20 min at 4°C with magnetic microbeads (Dynal Inc., Lake Success, NY) coated with anti-CD31 antibody. Cells bound to microbeads were magnetically captured and washed 5 times with binding buffer. Isolated cells were resuspended in 10% Endothelial Growth Medium-2 (EGM-2, Cambrex Bio Science Inc., Walkersville, MD) and incubated for 8–12 h at 37°C in 5% CO_2_/21% O_2_. The culture medium was changed every three days to remove unattached cells. Seven to 10 days after seeding the cells, the fast growing fibroblasts in the flask were repeatedly removed mechanically using the sharp end of a rubber rod under direct microscopy to allow visually identified PMVEC clusters to grow. When the PMVECs proliferated to 50% confluence, they were harvested with trypsin-EDTA and were re-purified with anti-CD31-coated magnetic microbeads as previously described. PMVEC were seeded in a 25-cm^2 ^flask and passaged at a ratio of 1:3. PMVEC were used for experiments at passages 2–3.

Inoculated PMVECs of the same concentration (10^6^/cm^2^) were divided into an Ad-ANXA1-transfected group, a non-transfected group, and an adenovirus empty vector group. Culture conditions were defined as 21% O_2 _and 5% CO_2_. When the cells covered 80% of the flask bottom, the original serum was replaced with 0.1% fetal calf serum. After 24 h of synchronous growth, the PMVECs were incubated under 5% HPS rat serum or normal rat serum conditions for 0 h (T1), 12 h (T2), and 24 h (T3). At 24 h prior to treatment, Ad-ANXA1, at a multiplicity of infection (MOI) of 60, was added into the medium of the Ad-ANXA1 transfection group. The flask of this group was placed upside-down on an inverted immunofluorescence microscope to observe the transfection efficiency.

### Real-time RT-PCR analysis

Total RNA was extracted at indicated time points using the Tripure Isolation Reagent. Reverse transcription (RT) was performed with 2 μg RNA, Oligo (dT)15 (Sangon, China), dNTP (Sangon, China), and the reaction buffer supplied with the M-MLV reverse transcriptase (Promega, USA). The fluorescence intensity ratio of the target gene to GAPDH was used as a measure of the relative gene expression. Specific primer sequences were used for the amplification of ANX A1 (forward primer: 5^′^- TAAGCGAAACAATGCACAGC −3^′^; reverse primer: 5^′^- CAAAGCGTTCCGAAAATCTC −3^′^; 331 bp) and GAPDH (forward primer: 5^′^-TGGAGTCCACTGGCGTCTTCAC-3^′^; reverse primers: 5^′^-CATGAGGTCCACCAGCCCTGTTG-3^′^; 698 bp).

### Western blotting

Standard techniques were used to collect cells. Cells were lysed in a buffer (0.5% Nonidet P-40, 10 mM Tris, pH 7.4, 150 mM NaCl, 1 mM EDTA, and 1 mM Na_3_VO_4_) containing a protease inhibitor (1 mM PMSF). The BCA protein assay was used to quantify protein. Equal quantities of protein were analyzed by sodium dodecyl sulfate-polyacrylamide gel electrophoresis (SDS-PAGE). The protein was transferred to the PVDF membranes. The membranes were blocked for 1 h using 5% milk in Tris-buffered saline with 0.1% Tween. After washing twice for 5 min each, the membranes were incubated with primary antibody (1:1000 dilution) at 37°C for 1 h and then overnight at 4°C. The membrane was washed twice with TBS, and then the bound antibody was detected using the IgG-HRP secondary antibody (1:500 dilution) for 30 min. The membranes were stained with diaminobenzidine, scanned, and stored using a gel imaging system. The optical density of the immunoreactivity was measured and analyzed with an Alpha Imager.

### Thymidine incorporation assay

After the cells of each group were digested, the solution of each group was made into a cell suspension to count the cell number. The cell concentration was adjusted to 2 × 10^4^/ml. Each group of cells was inoculated on a 96-well plate. The cells were cultured for 2 d prior to hypoxic treatment, and 1 μCi ^3^H-TdR (1 μCi/well) was added to each well 6 h prior to culture termination. The incorporation was stopped with cold PBS solution, and 0.25% trypsin was added to digest the cells and separate them from the cell wall. Cells were collected on a glass fiber filter with a multi-head harvester. The cells were washed with physiological saline 3 times, stabilized with 10% trichloroacetic acid, decolorized with absolute ethanol, dried for 30 min at 80°C, transferred into scintillation fluid, and counted with a liquid scintillation counter (counts/min, cpm).

### MTT assay

Cells (10^3^ - 10^4^) were inoculated into each well of a 96-well plate. The plate was placed in 5% CO_2_ and cultured for 24 h. Each group received the appropriate treatment for its specific experimental condition. The plate was cultured for an additional 24 h after replacing the medium with a serum-free medium. Next, 20 μl MTT solution (5 mg/ml) was added, and the plate was incubated for 4 h at 37°C. The supernatant from each well was then carefully suctioned away and discarded. Finally, 150 μl DMSO was added to each well, and the crystals were fully dissolved following 10 min of agitation. The absorbance value of each well was measured using an ELISA reader with 490 nm as the test wavelength and 280 nm as the reference wavelength.

### Statistical analysis

All data are expressed as the mean ± SEM*.* Comparisons between groups were carried out using Student’s *T* test or a Mann–Whitney *U*-test for parametric or non-parametric tests, respectively. The results were considered statistically significant when *p* < 0.05.

## Results

### CBDL rat serum decreased the transcript and protein expression levels of ANX A1 in PMVECs

**Figure 1:**
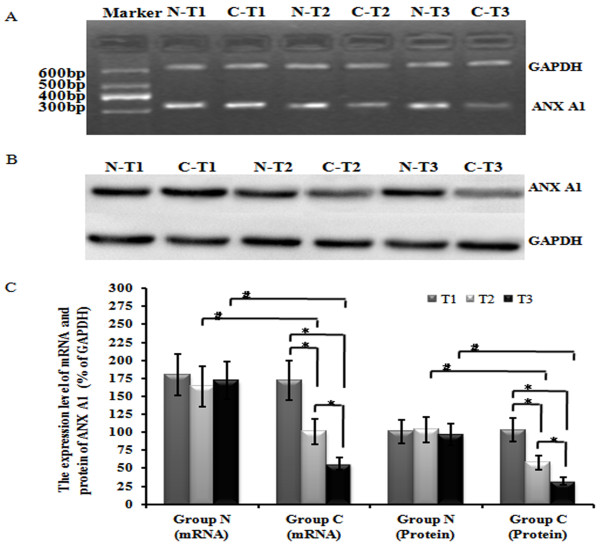
**Levels of ANX A1 mRNA and protein in PMVECs induced by CBDL-Rat serum at each time point.** (**A.B**) Exposure to CBDL-Rat serum significantly decreased expression of the ANX A1 gene, including the transcript and protein levels in PMVECs (*p* < 0.05). (**C**) The mRNA and protein density of the ANX A1 gene for each group is displayed. Each data point represents the mean ± S.E.M. of four independent experiments. * *p* < 0.05 vs. T1 or T2; # *p* < 0.05 vs. Group N. Group N: normal rat serum; Group C: CBDL-Rat serum.

ANX A1 transcript and protein expression levels were clearly detected in PMVECs of all groups. Following exposure to CBDL-rat-serum, the ANX A1 transcript and protein expression levels decreased persistently. There were significant differences between the CBDL-rat-serum group and normal-rat-serum group at T2 and T3 time points (*p* < 0.05). This finding indicates that CBDL rat serum down-regulates the expression level of the ANX A1 gene (Figure 1A, B and C).

### Ad-ANXA1 transfection was successful and the associated genes were strongly expressed in PMVECs

**Figure 2:**
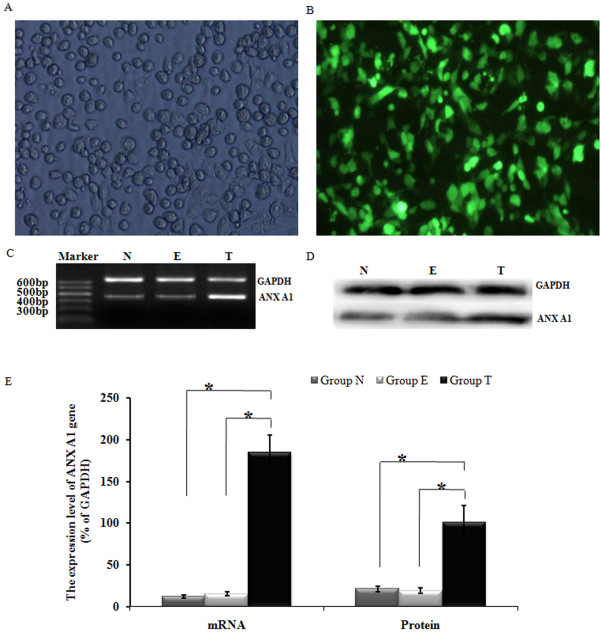
**Ad-ANXA1 transfected into PMVECs was strongly expressed and the protein was found in both the cytoplasm and nuclei.** (**A**) At 24 h after transfection with Ad-ANXA1, PMVECs had a normal structure with no observable pathological characteristics (magnification 100×). (**B**) Bright green fluorescence was observed in nearly all cells using an inverted fluorescence microscope following transfection with Ad-ANXA1 containing the green fluorescent protein (GFP) gene (magnification 100×). (**C.D**) The expression of the ANX A1 mRNA and protein in PMVECs 24 h after either the mock transfection or transfection with Ad-ANXA1 was determined by RT-PCR and western blotting. GAPDH was used as an internal control. (**E**) The mRNA and protein density of the ANX A1 gene for each group is displayed. Each data point represents the mean ± S.E.M. of four independent experiments. * *p* < 0.05; N: Non-transfected group; T: transfected group; E: empty vector group.

PMVECs appeared to have a normal structure and no pathological characteristics following transfection with a 60-MOI-dose of Ad-ANNXA1 for 24 h (Figure 2A). After transfection with Ad-ANNXA1 containing green fluorescent protein (GFP), cells were examined using an inverted fluorescence microscope. Bright green fluorescence was observed in the cytoplasm and nuclei of nearly all cells in the visual field (Figure 2B). At 24 h post-transfection with Ad-ANNXA1, the staining of ANNXA1 was easily observed in PMVECs after 12 h of treatment with HPS-rat-serum (Figure 2C). At 24 h after Ad-ANNXA1 transfection, a significant increase in both transcript (*p* < 0.05) (Figure 2D) and protein levels of ANNXA1 (*p* < 0.05) (Figure 2E) was observed. These findings demonstrate that over 95% of PMVECs were successfully transfected with Ad-ANNXA1 and that the genes encoded by Ad-ANNXA1 were effectively expressed as proteins in the cytoplasm and nuclei of PMVECs.

### Over-expression of ANX A1 suppressed CBDL-rat-serum-induced increase in the a1-actin, a1-tubulin and destrin protein expression levels in PMVECs

**Figure 3:**
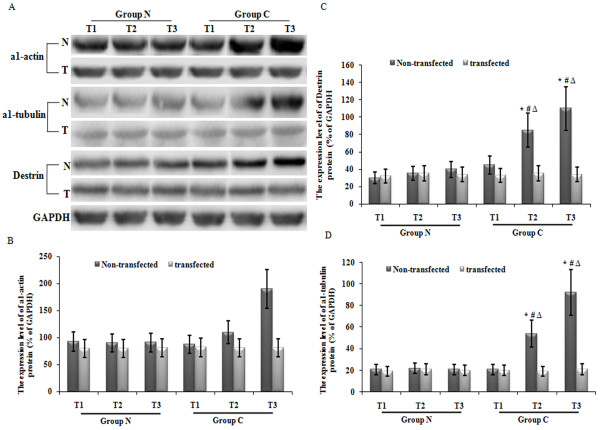
**Over-expression of ANX A1 suppressed CBDL-rat-serum-induced increase in the a1-actin, a1-tubulin and destrin protein expression levels in PMVECs.** (**A**) The change in expression levels of a1-actin, a1-tubulin and destrin proteins in PMVECs 24 h following either mock transfection or transfection with Ad-ANXA1 was determined by western blot. GAPDH was used as an internal control. (**B-D**) The protein density of a1-actin, a1-tubulin and destrin for each group is displayed. Each data point represents the mean ± S.E.M. of four independent experiments. * *p* < 0.05 vs. group N; ^#^*p* < 0.05 vs. the non-transfected group; Δ*p* < 0.05 vs. T1. N: Non-transfected; T: transfected with Ad-ANXA1.

Western blot analysis indicated that incubation in normal rat serum did not increase the a1-actin, a1-tubulin and destrin protein expression levels, which remained consistently low at each time point observed. Additionally, there was no significant difference among each time point (T1, T2 and T3) (p > 0.05). However, exposure to CBDL rat serum significantly increased a1-actin, a1-tubulin and destrin protein expression, and as the exposure time increased, the expression of a1-actin, a1-tubulin and destrin also increased (*p* < 0.05). After transfection of Ad-ANXA1, the expression of a1-actin, a1-tubulin and destrin induced by CBDL rat serum was inhibited significantly at each time point (T2 an d T3) (Figure 3).

### Over-expression of ANX A1 inhibited CBDL rat serum-induced proliferation of PMVECs identified with 3H-TdR incorporation assay and MTT assay

**Figure 4:**
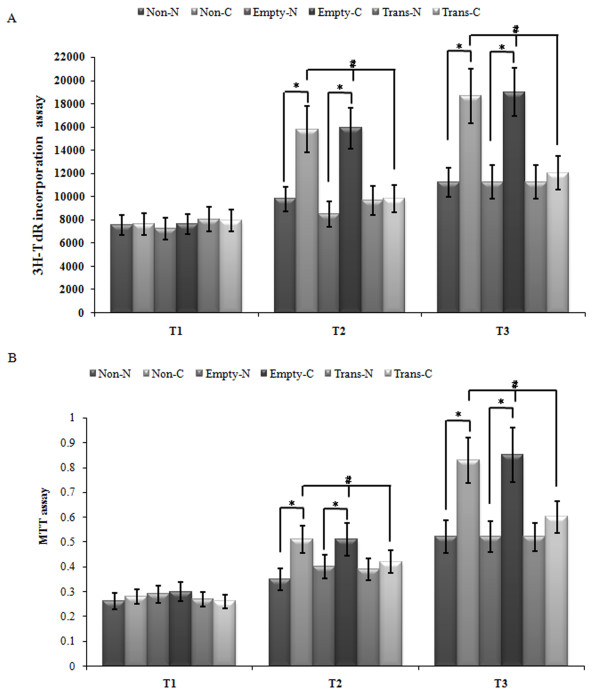
**Over-expression of the ANXA1 gene inhibited CBDL-rat-serum-induced PMVEC proliferation as determined by the **^**3**^**H-TdR and MTT assay.** (**A.B**) The change of the value of the incorporation of ^3^H-TdR and the absorbance of MTT assay is displayed. The inhibitory effect was investigated at T2 and T3. Each data point represents the mean ± S.E.M. of four independent experiments. * *P* < 0.05 vs. group N; # *P* < 0.05 vs. Non-transfected group and empty vector group. Non-N: Group N without Ad-ANXA1 transfection; Non-C: Group C without Ad-ANXA1 transfection; Empty-N: Group N with empty vector transfection; Empty-C: Group C with empty vector transfection; Trans-N: Group N with Ad-ANXA1 transfection; Trans-C: Group C with Ad-ANXA1 transfection.

Under normal rat serum conditions, PMVECs exhibited relatively low ^3^H-TdR incorporation. Following exposure to CBDL rat serum, ^3^H-TdR incorporation increased gradually as exposure time increased (*p* < 0.05). CBDL rat serum enhanced rat PMVEC proliferation by more than two-fold after 24 h of exposure in the non-transfected (*p* < 0.05) and the empty vector groups. Incorporation of ^3^H-TdR in the transfected group, however, was significantly inhibited compared to the non-transfected or the empty vector group. Differences in the incorporation of ^3^H-TdR were significant at each time point measured (T1, T2, and T3) (*p* < 0.05) (Figure 4A).

The absorbance of MTT in each group increased gradually as the exposure time to CBDL rat serum increased (*p < 0.05*). The absorbance of MTT in the transfected group was significantly inhibited compared to both the non-transfected and the empty vector group. Differences in the absorbance of MTT were significant (p < 0.05) at each time point measured (T1, T2, and T3) (Figure 4B).

## Discussion

Liver cirrhosis and portal hypertension can result in pulmonary vascular remodeling, which may involve changes in cytokine signaling, leading to altered gene expression. However, the cellular signaling mechanisms involved in HPS–associated pulmonary vascular remodeling are still poorly understood. Previous studies and our initial research have demonstrated that the differentiation and proliferation of PMVECs play an important role in the pathogenesis of IPVD of HPS and that cytoskeletal remodeling is the key change in the pathophysiology of the proliferation or differentiation of PMVECs [1,3,18,19]. Whether the regulation of cytoskeletal proteins in PMVECs is a good therapeutic approach demands further investigation. The major finding of this study is that the ANX A1-mediated regulation of cytoskeletal protein expression might play an important role in the proliferation of rat PMVECs induced by CBDL-Rat serum and that ANX A1 might be one new therapeutic target for the development of IPVD. The critical protein involved in the development of HPS requires further research. Our work is the first to demonstrate the role of ANX A1 and cytoskeletal proteins in the proliferation and differentiation of PMVECs induced by CBDL-Rat serum.

Our previous study showed that CBDL-Rat serum induced the proliferation and differentiation of PMVECs [18,19]. The proliferation of PMVECs has been previously shown to be accompanied by changes in cytoskeleton remodeling and cytoskeletal protein expression changes. Dynamic regulation of the cytoskeleton is critical for cell motility, differentiation and proliferation due to a variety of factors. During this process, actin stress fibers are first depolymerized, and cortical actin is re-polymerized at the leading edge of the cell. Our study showed that expression of cytoskeletal proteins, including a1-actin, a1-tubulin and destrin, in rat PMVECs was up-regulated significantly under the CBDL-Rat serum condition. This result suggested that CBDL-Rat serum could promote the cytoskeletal remodeling of PMVECs, which might be a key change of PMVECs in IPVD. However, the detailed circumstances and molecular mechanisms have not been defined.

Our data indicated that the expression of ANX A1 in PMVECs decreased clearly under the CBDL-Rat serum condition. Further, the over-expression of ANX A1 by transfection of Ad-ANXA1 could reverse the proliferation of PMVECs induced by the CBDL-Rat serum. This finding suggests that ANX A1 might play the role of an inhibitor in the process of PMVEC proliferation. Furthermore, we found that the over-expression of ANX A1 by transfection of Ad-ANXA1 reversed the up-regulation of a1-actin, a1-tubulin and destrin in PMVECs induced by CBDL-Rat serum. This finding suggests that ANX A1 might be an inhibitor during the process of PMVEC differentiation.

Interestingly, we have reported that ANX A1 is activated during hypoxia-induced PASMC migration, which was accompanied by the down-regulated expression of several cytoskeletal proteins, including SM-α-actin, MHC and calponin, and cell migration in our initial study [22]. Thus, ANX A1 might be a key regulator of cytoskeleton remodeling in PASMCs and PMVECs. Because both PASMCs and PMVECs are involved in the pathogenesis of IPVD, ANX A1 might be a more suitable therapeutic target in HPS. However, more research is required.

## Conclusion

In conclusion, our findings indicate that ANX A1 plays an important regulatory role in cytoskeleton remodeling of PMVECs in the pathogenesis of HPS. In the future, it will be useful to understand the mechanisms of abnormal cell proliferation and differentiation, as well as the endogenous regulatory machinery in the cytokine signaling cascade, to provide a basis for circumventing the process and creating targeted therapies for diseases associated with vascular remodeling in HPS.

## Competing interests

The authors declare that they have no competing interests.

## Authors’ contributions

BY, GSQ and KZL conceived and designed the experiments. BY and JZ Performed the experiments. BY, GSW and GSQ analyzed the data. BY, JZ, GSW, GSQ and KZL contributed reagents, materials or analysis tools. BY and JZ wrote the paper. All authors read and approved the final manuscript.
